# Supplementary material for: The essential role of PRAK in tumor metastasis and its therapeutic potential
Source: Nat Commun. 2021 Mar 19;12:1736. doi: 10.1038/s41467-021-21993-9 (PMC7979731; doi:10.1038/s41467-021-21993-9)
Supplement: Supplementary file 1 — Supplementary Information [file 41467_2021_21993_MOESM1_ESM.pdf]

The essential role of PRAK in tumor metastasis and its therapeutic potential.

Yuqing Wang[a,b,#], Wei Wang[a,#], Haoming Wu[a], Yu Zhou[a], Xiaodan Qin[a], Yan Wang[a], Jia Wu[a], Xiu-Yuan Sun[a], Yan Yang[c], Hui Xu[c], Xiaoping Qian[a], Xuewen Pang[a], Yan Li[a], Zhiqian Zhang[d], Jiahuai Han[e] and Yu Zhang[a,c,*]

[a] Department of Immunology, School of Basic Medical Sciences, Peking University. NHC Key Laboratory of Medical Immunology (Peking University), Beijing, 100191,China.

[b] Peking University Third Hospital, Center of Basic Medical Research, Institute of Medical Innovation and Research, Beijing, China.

[c] Institute of Biological Sciences, Jinzhou Medical University, Jinzhou, Liaoning, China

[d] Key laboratory of Carcinogenesis and Translational Research (ministry of Education), Department of Cell Biology, Peking University Cancer Hospital and Institute, Beijing, China

[e] State Key Laboratory of Cellular Stress Biology, Innovation Center for Cell Signaling Network, School of Life Sciences, Xiamen University, Xiamen, Fujian, China

#These authors contribute equally to this work.

*Correspondence and requests for materials should be addressed to Yu Zhang, Department of Immunology, Peking University Health Science Center, 38 Xue Yuan Road, Beijing, 100191, China, Tel: 0086-10-82802593, Fax: 0086-10-82801436, E-mail: zhangyu007@hsc.pku.edu.cn

Supplemental information

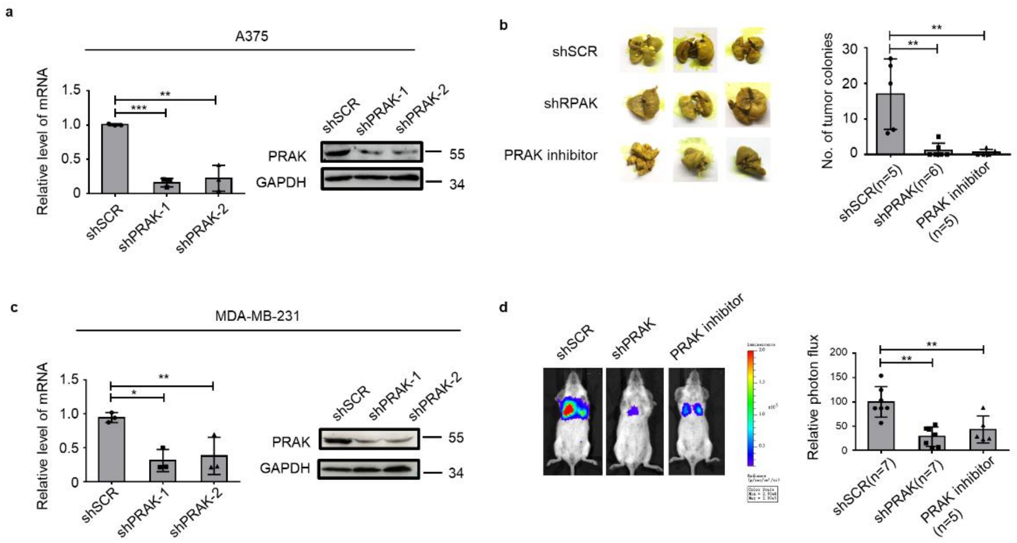

Supplementary Figure 1

(**a**) A375 cells were infected with lentiviruses carrying shPRAK or control scrambled shRNA (shSCR). The knockdown efficiency was validated by real-time RT-PCR repeated 3 times with triplicates (left) and western blotting (right). Bars are the mean and error bars represent s.e. $**p < 0.01$, $***p < 0.001$. (**b**) A375 cells carrying shPRAK or shSCR were intravenously injected into SCID mice. The PRAK inhibitor-treated group received daily intraperitoneal injection of inhibitor at 2mg/kg from day 0 to 4. Tumor colonies in the lung was examined at day 29. Representative images are shown on the left. Data collected from 5-6 mice in each group are presented as mean ± s.d. $**p < 0.01$. (**c**) MDA-MB-231-Luc-D3H2LN cells were infected with lentiviruses carrying shPRAK or shSCR. The knockdown efficiency was validated by real-time RT-PCR repeated 3 times with triplicates (left) and western blotting (right). Bars are the mean and error bars represent s.e. $*p < 0.05$, $**p < 0.01$. (**d**) MDA-MB-231-Luc-D3H2LN cells carrying shPRAK or shSCR were intravenously injected into SCID mice. The PRAK inhibitor-treated group received daily intraperitoneal injection of inhibitor at 2mg/kg from day 0 to 4. Lung metastasis was monitored by bioluminescent imaging at day 21. Representative images are shown on the left. Data collected from 5-7 mice in each group are presented as mean ± s.d. $**p < 0.01$. p-value was determined by two-tailed, unpaired t-test.

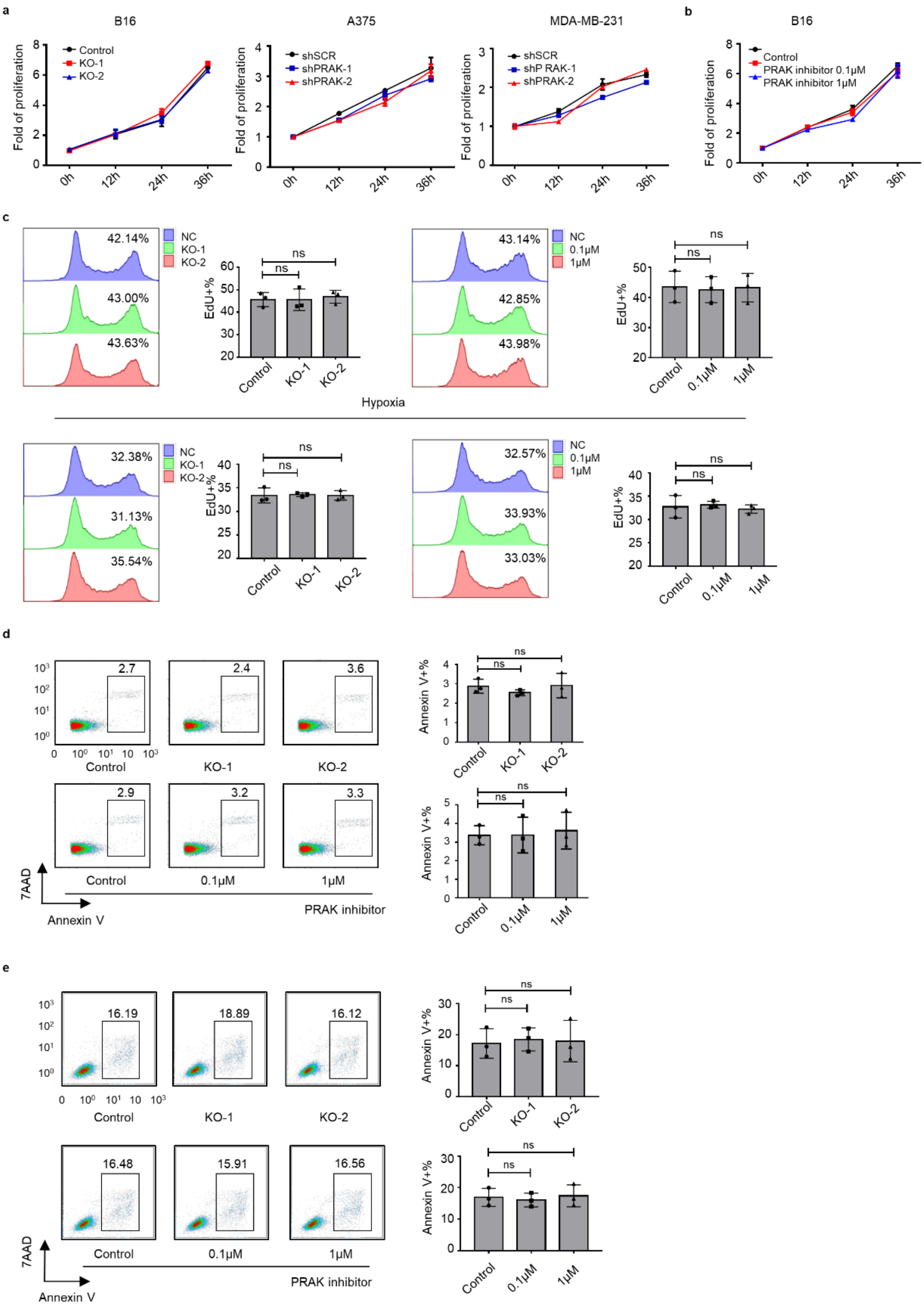

a
B16
A375
MDA-MB-231
Fold of proliferation
Control
KO-1
KO-2
shSCR
shPRAK-1
shPRAK-2
shP RAK-1
0h
12h
24h
36h
b
B16
PRAK inhibitor 0.1μM
PRAK inhibitor 1μM
c
NC
0.1μM
1μM
42.14%
43.00%
43.63%
43.14%
42.85%
43.98%
EdU+%
ns
Hypoxia
32.38%
31.13%
35.54%
32.57%
33.93%
33.03%
d
2.7
2.4
3.6
2.9
3.2
3.3
Annexin V+%
7AAD
Annexin V
PRAK inhibitor
e
16.19
18.89
16.12
16.48
15.91
16.56

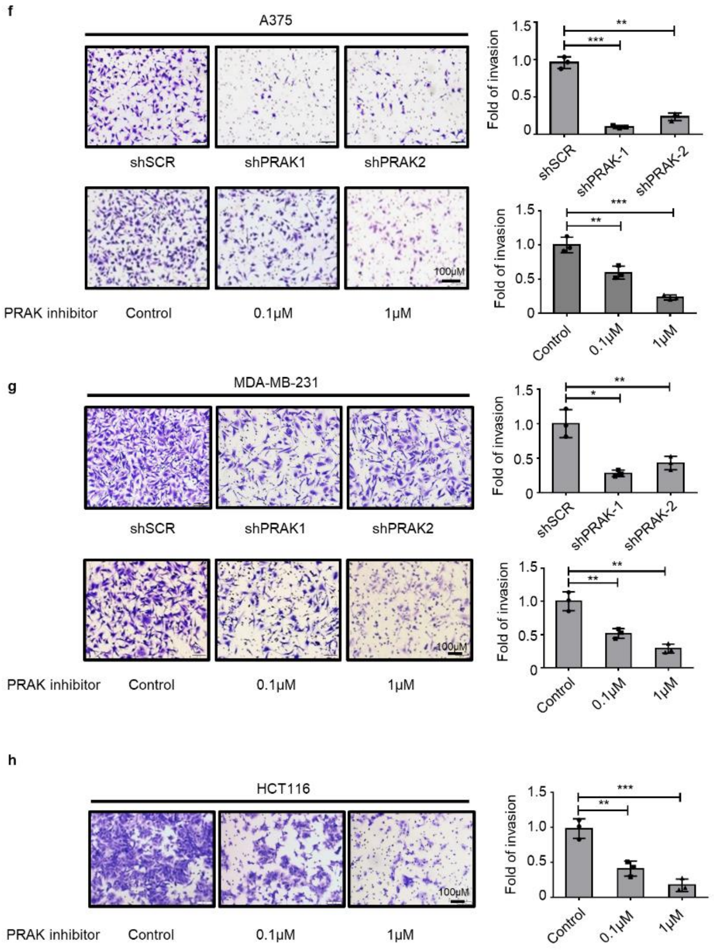

Supplementary Figure 2

(**a-b**) MTS assay was performed to analyze the proliferation of *Prak* knockout B16, *Prak* knockdown A375 and MDA-MB-231-Luc-D3H2LN cells and control cells (**a**) and that of B16 cells in the presence or absence of the PRAK inhibitor (**b**). Data are presented as mean ± s.e. from 3 independent experiments with triplicates for each time point. ns, not significant. (**c**) Proliferation of WT and KO B16 cells and that of wild type cells in the presence or absence of the PRAK inhibitor was also examined by EdU incorporation (upper). Similar experiments were performed under hypoxic conditions (lower). Data are presented as mean ± s.e. from 3 independent experiments with triplicates for each time points. ns, not significant. (**d&e**) Apoptosis of *Prak*$^{+/+}$ and *Prak*$^{-/-}$ B16 cells and cells treated with the PRAK inhibitor as measured by AnnexinV/7AAD staining (**d**). Similar experiments were performed after incubation with cisplatin for 24 hrs (**e**). Representative dot blots are shown on the left. Data

collected from 3 independent experiments are presented as mean ± s.d. ns, not significant. (**f**) Matrigel invasion assay for *Prak* knockdown and inhibitor-treated A375 cells. The experiments were repeated 3 times. Representative images and quantification of fold of invasion are shown. Error bars represent s.d. Scale bars, 100 µm. **$p < 0.01$, ***$p < 0.001$. (**g**) Matrigel invasion assays for *Prak* knockdown and inhibitor-treated MDA-MB-231-Luc-D3H2LNc cells. Data are presented as mean ± s.e. from 3 independent experiments with triplicates for each time point. ns, not significant. *$p < 0.05$, **$p < 0.01$. (**h**) Matrigel invasion assays for PRAK inhibitor-treated HCT-116 cells. Data are presented as mean ± s.e. from 3 independent experiments with triplicates for each time point. ns, not significant. **$p < 0.01$, ***$p < 0.001$. p-value was determined by two-tailed, unpaired t-test

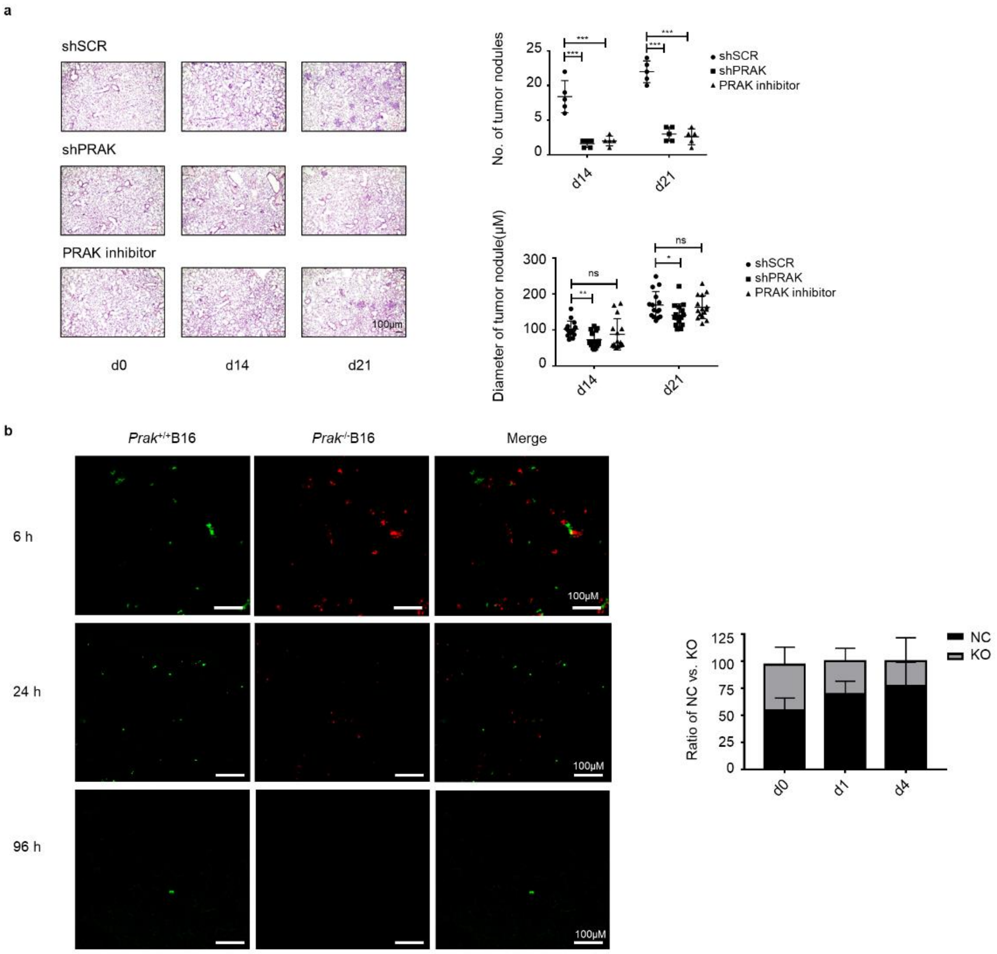

Supplementary Figure 3

(**a**) MDA-MB-231-Luc-D3H2LN cells carrying shPRAK or shSCR were intravenously injected into SCID mice (n=3 for each time point in each group). The PRAK inhibitor-treated group received daily intraperitoneal injection of inhibitor at 2mg/kg from day 0 to 4. The lung tissue was harvested at different time points and examined for the presence of tumor colonies after H&E staining. Representative images are shown in the upper panel. Scale bars, 100μm. The average number of tumor nodules in 5 randomly selected fields and the diameter of the nodules are shown in the middle and lower panel, respectively. Bars are the mean and error bars represent s.d. *p < 0.05, **p < 0.01, ***p < 0.001, ns, not significant. (**b**) Equal numbers of *Prak*$^{+/+}$ (green) and *Prak*$^{-/-}$ (red) B16 cells were mixed and injected into the mouse through the tail vein. Colonization of tumor cells in the pulmonary parenchyma was examined using confocal microscopy. Representative images from 3 independent experiments are shown at hour 6, 24 and 96 post-injections. The ratio of

Prak$^{+/+}$ (WT) and *Prak*$^{-/-}$ (KO) were calculated based on cell counts from 5 randomly slected fields. Bars are the mean and error bars represent s.d. p-value was determined by two-tailed, unpaired t-test

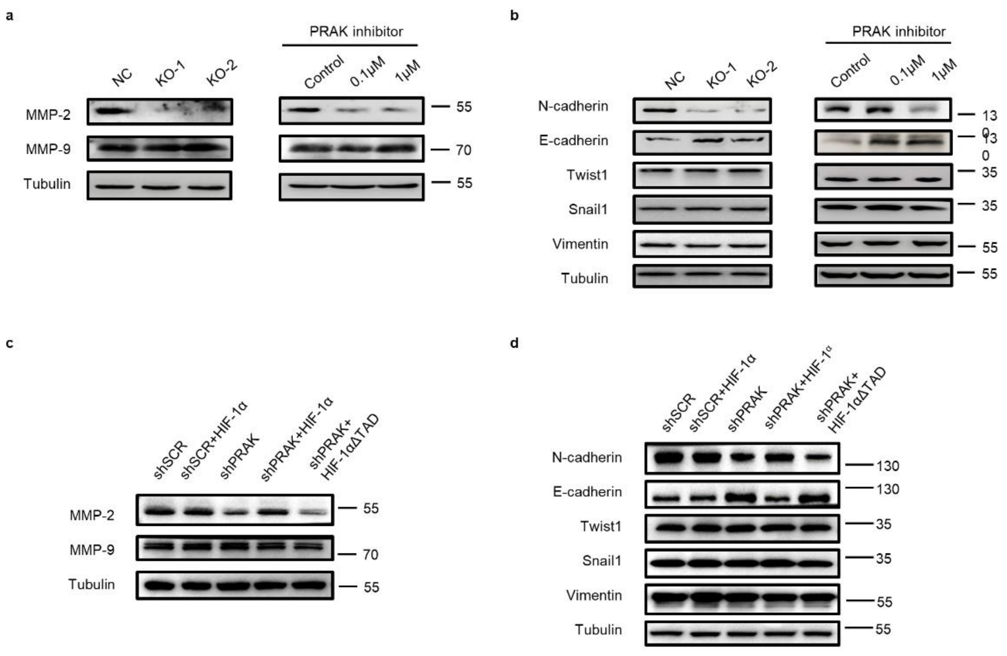

Supplementary Figure 4

(**a-b**) Western blotting analysis of MMP2 and MMP9 (**a**) and EMT signature molecule (**b**) expression in parent, *Prak* knockout, or inhibitor-treated B16 cells. The experiments were repeated 3 times with similar results. (**c-d**) MDA-MB-231 cells were infected with lentiviruses carrying shSCR, shSCR+HIF-1α, shPRAK, shPRAK+HIF-1α, or shPRAK+ HIF-1αΔTAD. Expression of MMP2 and MMP9 (**c**) and EMT signature molecules (**d**) was examined by Western blot analysis. The experiments were repeated 2 times with similar results.

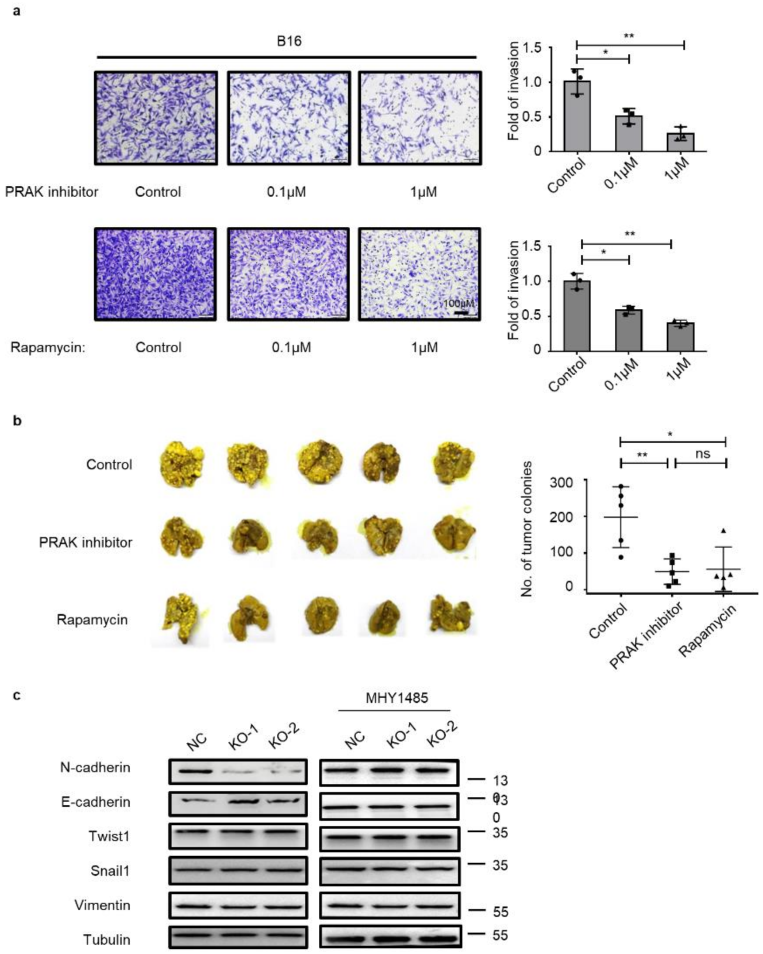

Supplementary Figure 5

(**a**) *Prak*$^{+/+}$ and *Prak*$^{-/-}$ B16 cells were cultured with or without the mTORC1 agonist MHY1485 (1μM for 12 hours). EMT signature molecule expression was detected by immunoblotting. (**b**) B16 cells were treated with the PRAK inhibitor GLPG0259 and the mTORC1 inhibitor rapamycin. Their invasive capacity was analyzed using Matrigel assay. The experiments were repeated 3 times. Representative images and quantification of fold of invasion are shown. Error bars represent s.d. Scale bars, 100 μm. *$p < 0.05$, **$p < 0.01$, ***$p < 0.001$. (**c**) MCF7 cells were intravenously injected into SCID mice. The PRAK inhibitor- and rapamycin-treated group received daily intraperitoneal injection of inhibitor at 2mg/kg from day 0 to 4. Tumor colonies in the lung was examined at day 30. Representative images are shown on the left. Data collected from 5 mice in each group are presented as mean ± s.d. *$p < 0.05$, **$p < 0.01$, ns, not significant. p-value was determined by two-tailed, unpaired t-test

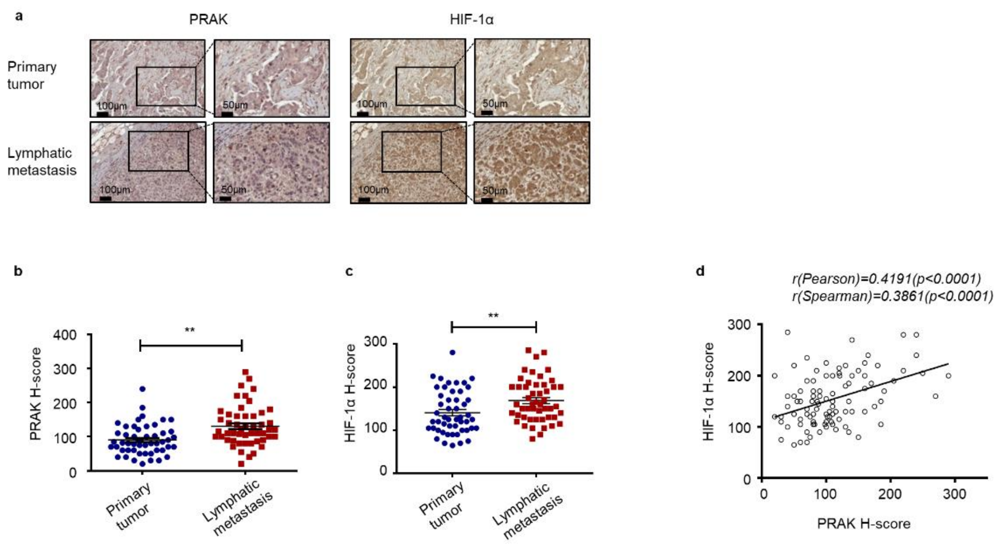

Supplementary Figure 6

A human tissue array containing 40 breast carcinoma samples with paired lymphatic metastasis were analyzed for PRAK and HIF-1α expression by immunohistochemistry. (**a**) Representative images of PRAK and HIF-1α staining of primary tumors and lymphatic metastases. (**b**) H-scores for PRAK expression in primary tumors versus lymphatic metastases. Each symbol represents an individual sample (n=50 for each group). Bars are the mean and error bars represent s.e. **$p < 0.01$. (**c**) H-scores for HIF-1α expression in primary tumors versus lymphatic metastases (n=50 for each group). **$p < 0.01$. (**d**) The correlation of PRAK and HIF-1α expression as revealed by R programming. p-value was determined by paired t test followed by Wilcoxon text (**b,c**).

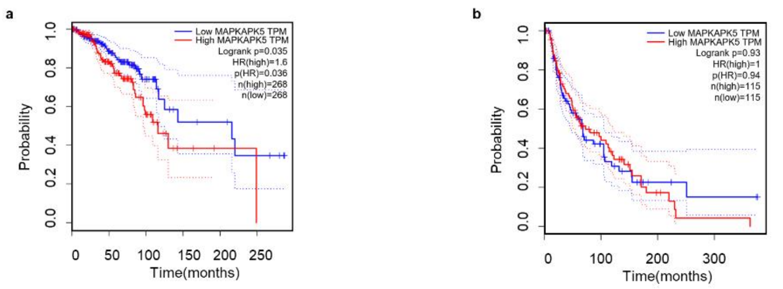

Supplementary Figure 7

Survival analysis was performed with a cohort of 536 breast cancer patients (**a**) and 230 skin melanoma patients (**b**) from a public database (http://gepia.cancer-pku.cn/) to explore the correlation of overall survival with relative expression levels of PRAK.

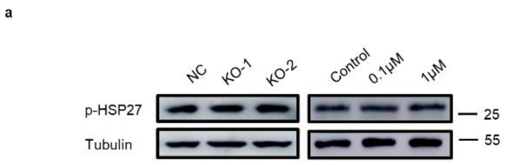

Supplementary Figure 8

(**a**) The phosphorylation of HSP27 in parent, *Prak* knockout, and PRAK inhibitor-treated B16 cells was determined by Western blotting.

Supplementary Table 1. List of primers used in the study.

Primer	Sequence 5’-3’
HIF-1α-F	ACCTTCATCGGAAACTCCAAAG
HIF-1α-F	CTGTTAGGCTGGGAAAAGTTAGG
PRAK-F	CCAGCACCGGCACTTTACA
PRAK-R	TGGTCAATCTTGGCAAATCCAAA
β-actin-F	TATGGAATCCTGTGGCATC
β-actin-R	GTGTTGGCATAGAGGTCTT
