# Supplementary material for: The essential role of PRAK in tumor metastasis and its therapeutic potential
Source: Nat Commun. 2021 Mar 19;12:1736. doi: 10.1038/s41467-021-21993-9 (PMC7979731; doi:10.1038/s41467-021-21993-9)
Supplement: Supplementary file 3 — Source Data [file 41467_2021_21993_MOESM3_ESM.pdf]

Figure 1C

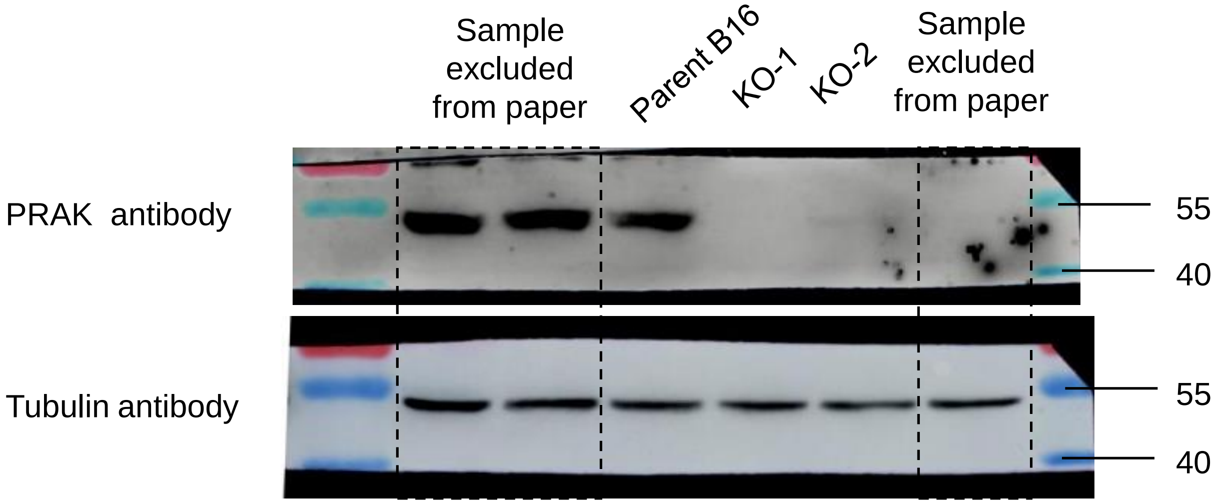
Sample excluded from paper
Parent B16
KO-1
KO-2
Sample excluded from paper
PRAK antibody
55
40
Tubulin antibody
55
40

Figure 2D

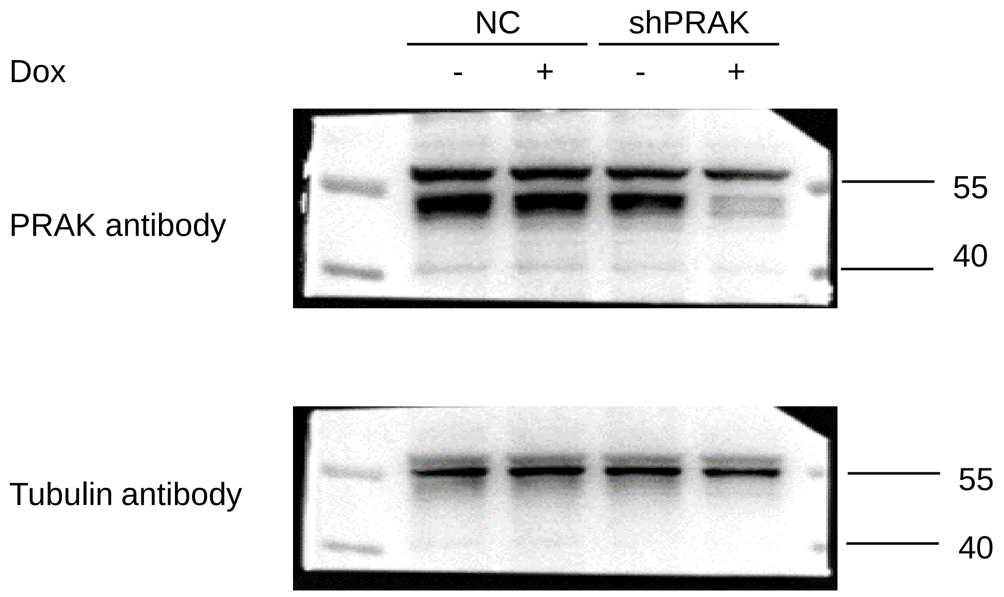

Figure 4D

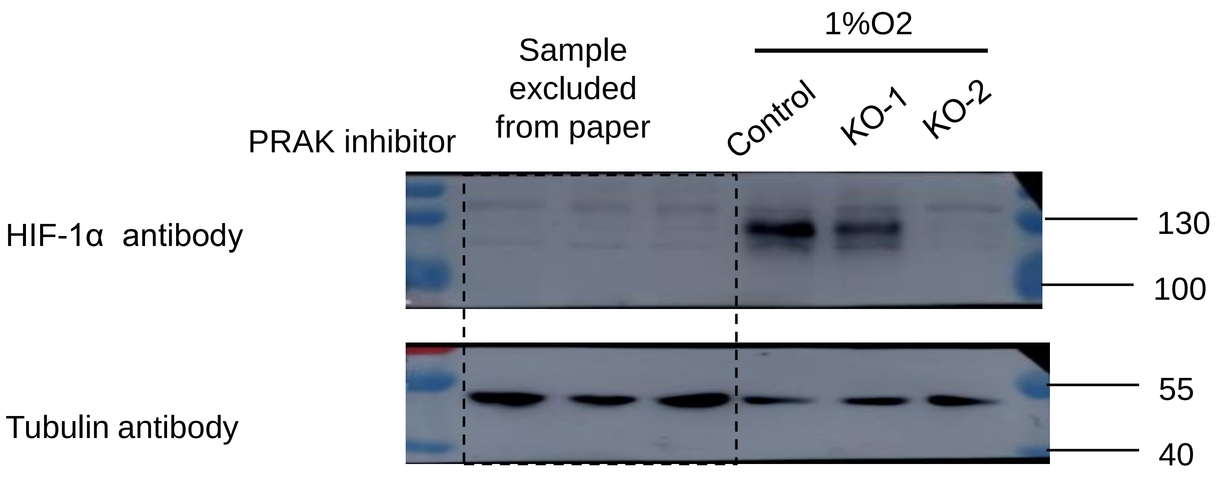

PRAK inhibitor
Sample excluded from paper
1%O2
Control
KO-1
KO-2
HIF-1α antibody
Tubulin antibody
130
100
55
40

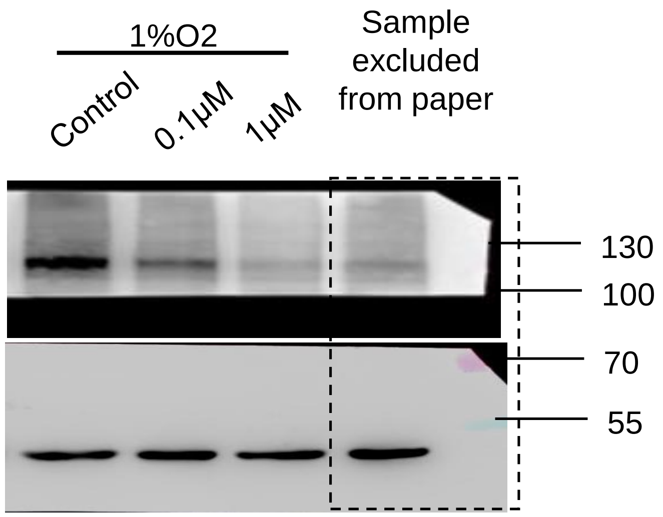

1%O2
Control
0.1μM
1μM
Sample excluded from paper
130
100
70
55

Figure 4E

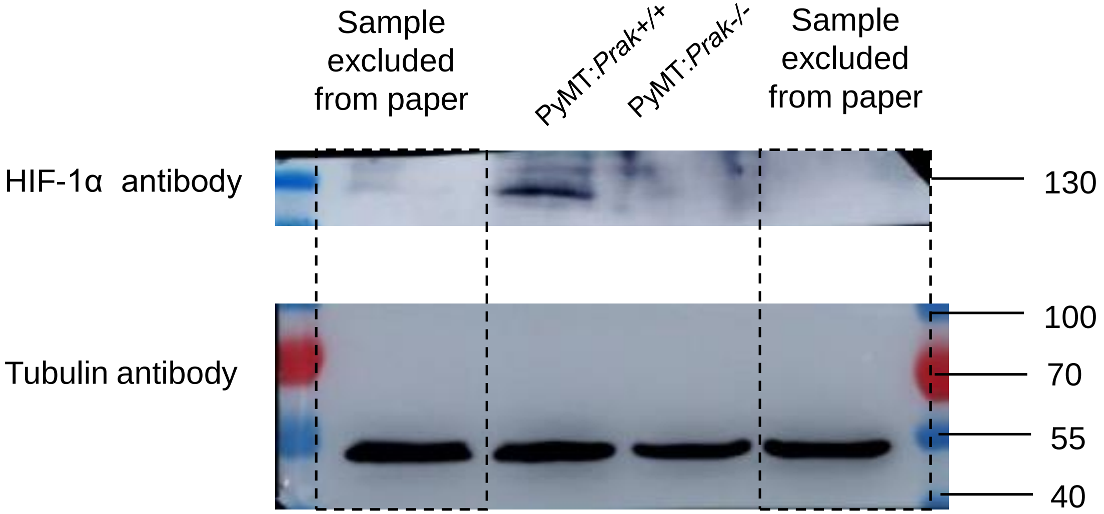

Sample excluded from paper
PyMT:Prak+/+
PyMT:Prak-/-
Sample excluded from paper
HIF-1α antibody
Tubulin antibody
130
100
70
55
40

Figure 4F

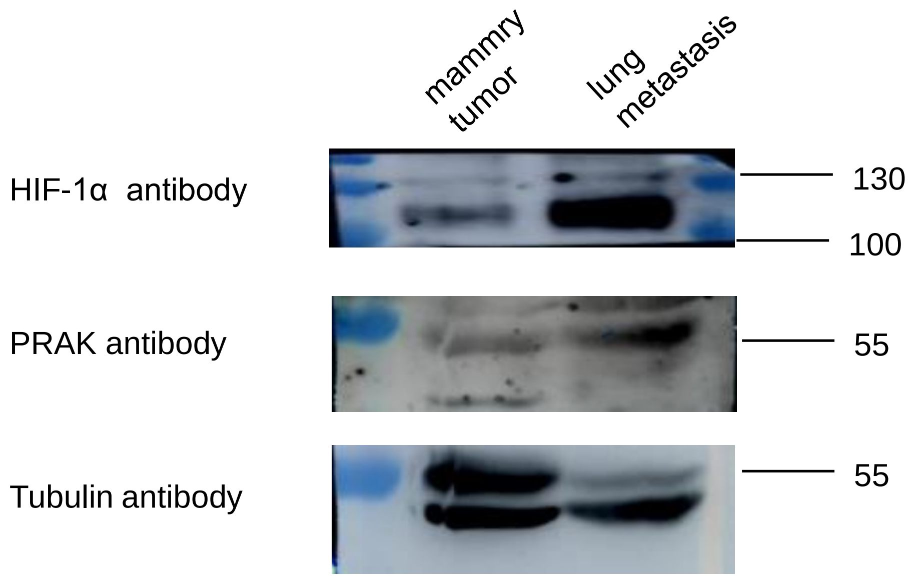

mammry tumor
lung metastasis
HIF-1α antibody
PRAK antibody
Tubulin antibody
130
100
55
55

Figure 5A

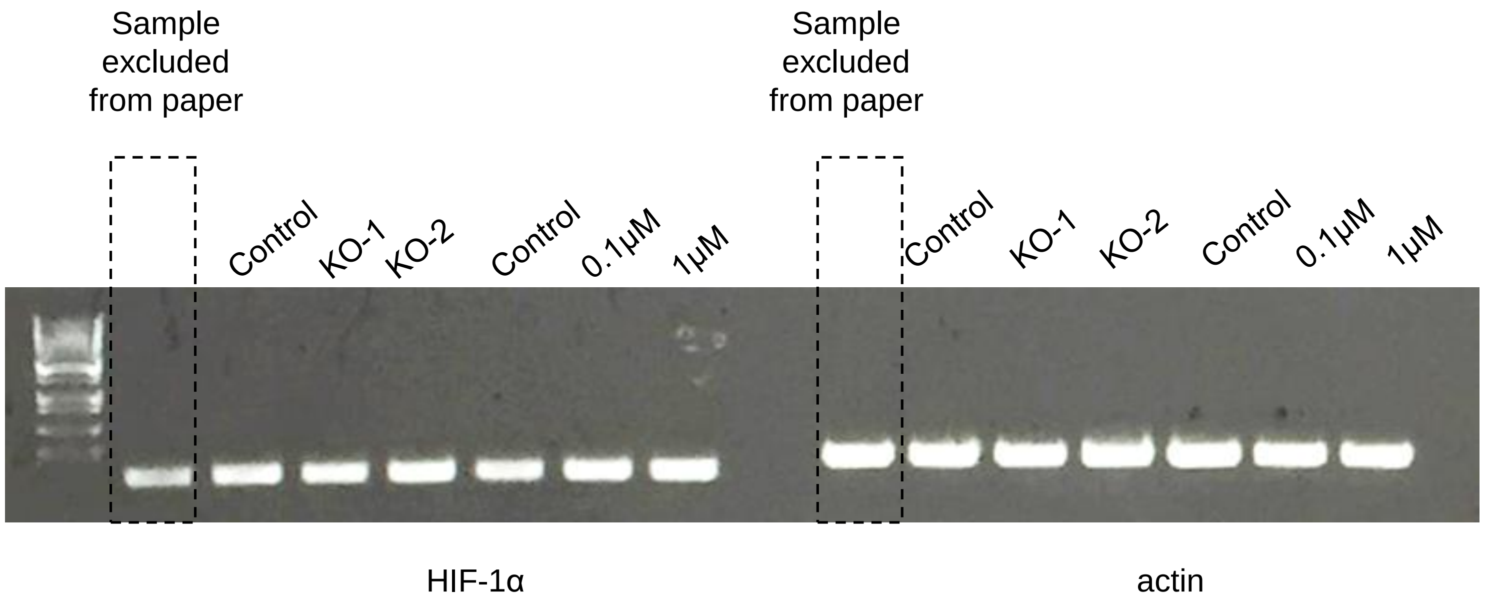

Sample excluded from paper
Control
KO-1
KO-2
Control
0.1μM
1μM
Sample excluded from paper
Control
KO-1
KO-2
Control
0.1μM
1μM
HIF-1α
actin

Figure 5B

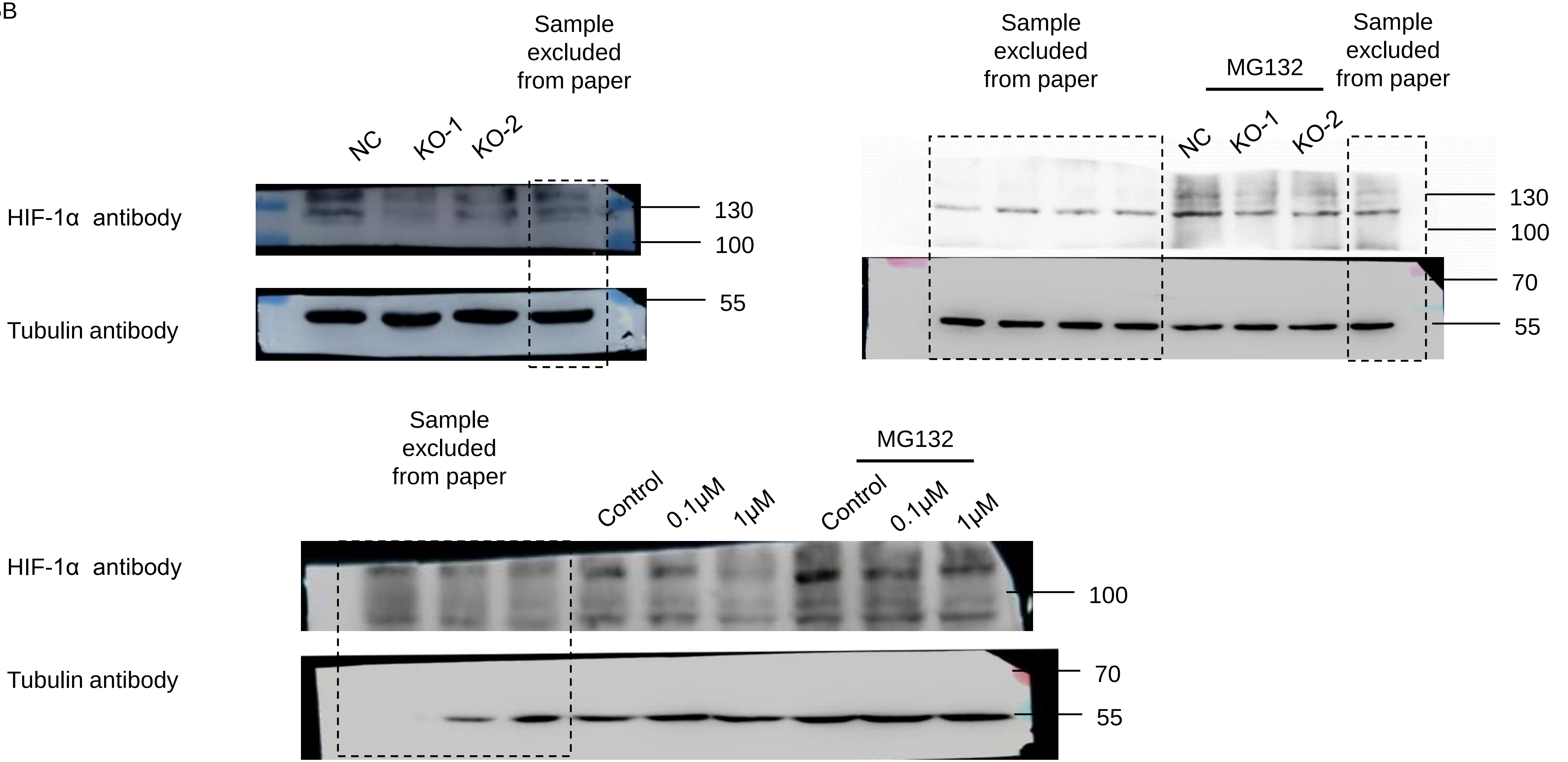
Sample excluded from paper
NC
KO-1
KO-2
HIF-1α antibody
130
100
Tubulin antibody
55
Sample excluded from paper
MG132
Sample excluded from paper
NC
KO-1
KO-2
130
100
70
55
Sample excluded from paper
Control
0.1μM
1μM
MG132
Control
0.1μM
1μM
HIF-1α antibody
100
Tubulin antibody
70
55

Figure 5C

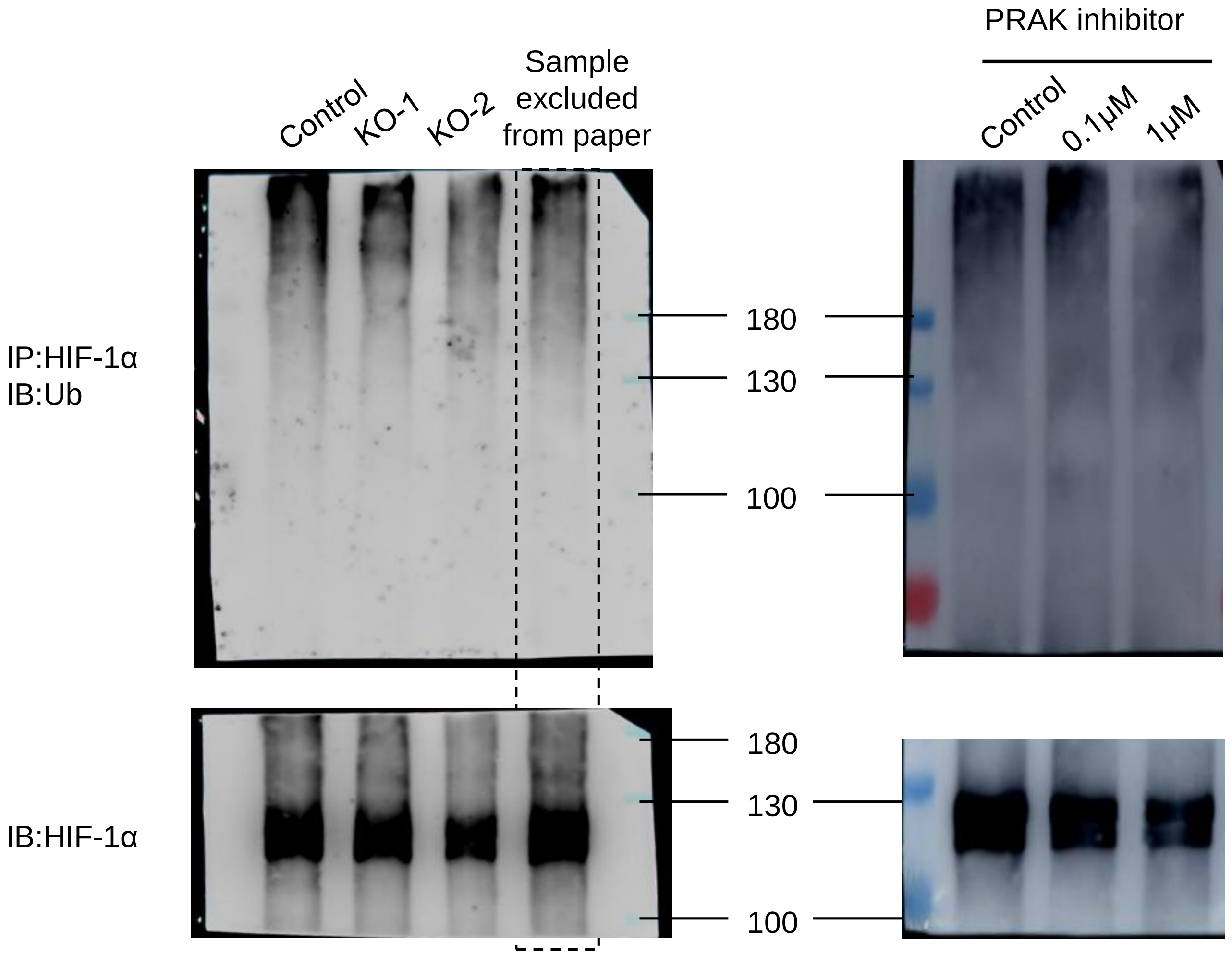
PRAK inhibitor
Control
KO-1
KO-2
Sample excluded from paper
Control
0.1μM
1μM
IP:HIF-1α
IB:Ub
180
130
100
IB:HIF-1α
180
130
100

Figure 5D

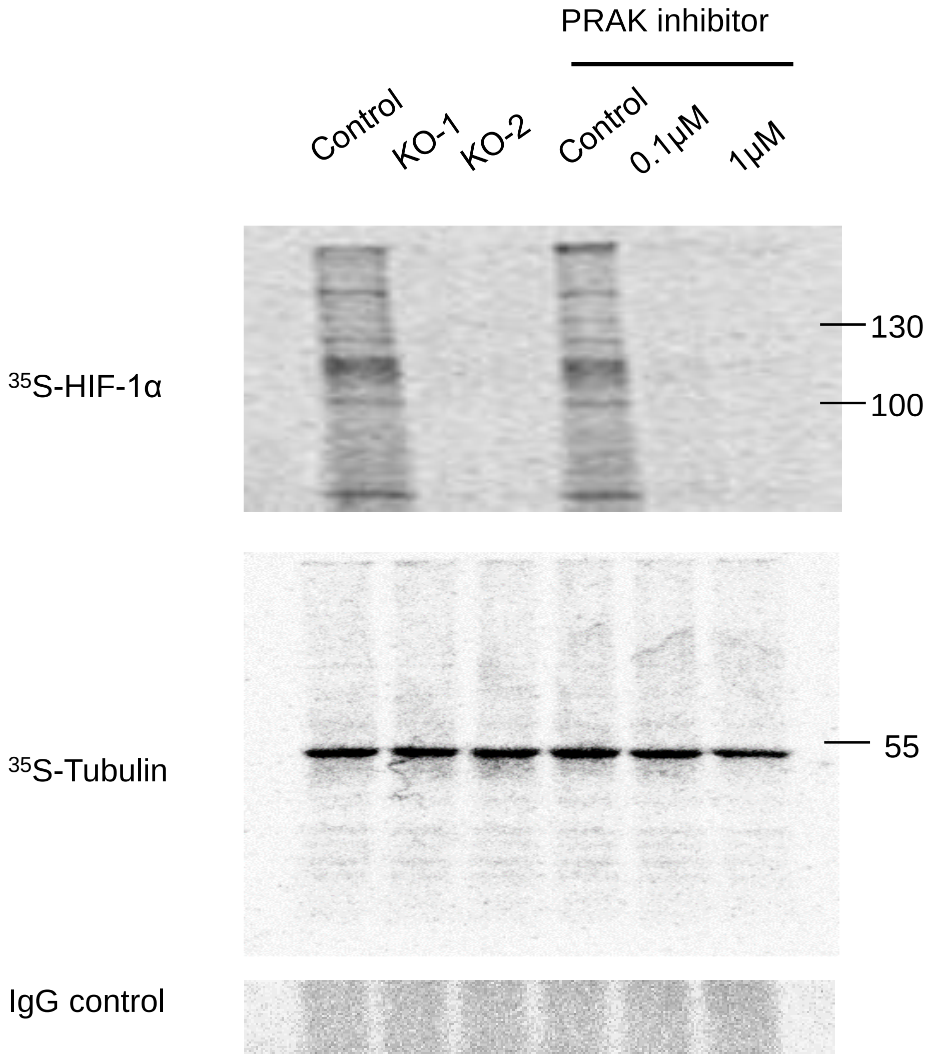
PRAK inhibitor
Control
KO-1
KO-2
Control
0.1μM
1μM
130
100
55
35S-HIF-1α
35S-Tubulin
IgG control

Figure 5E

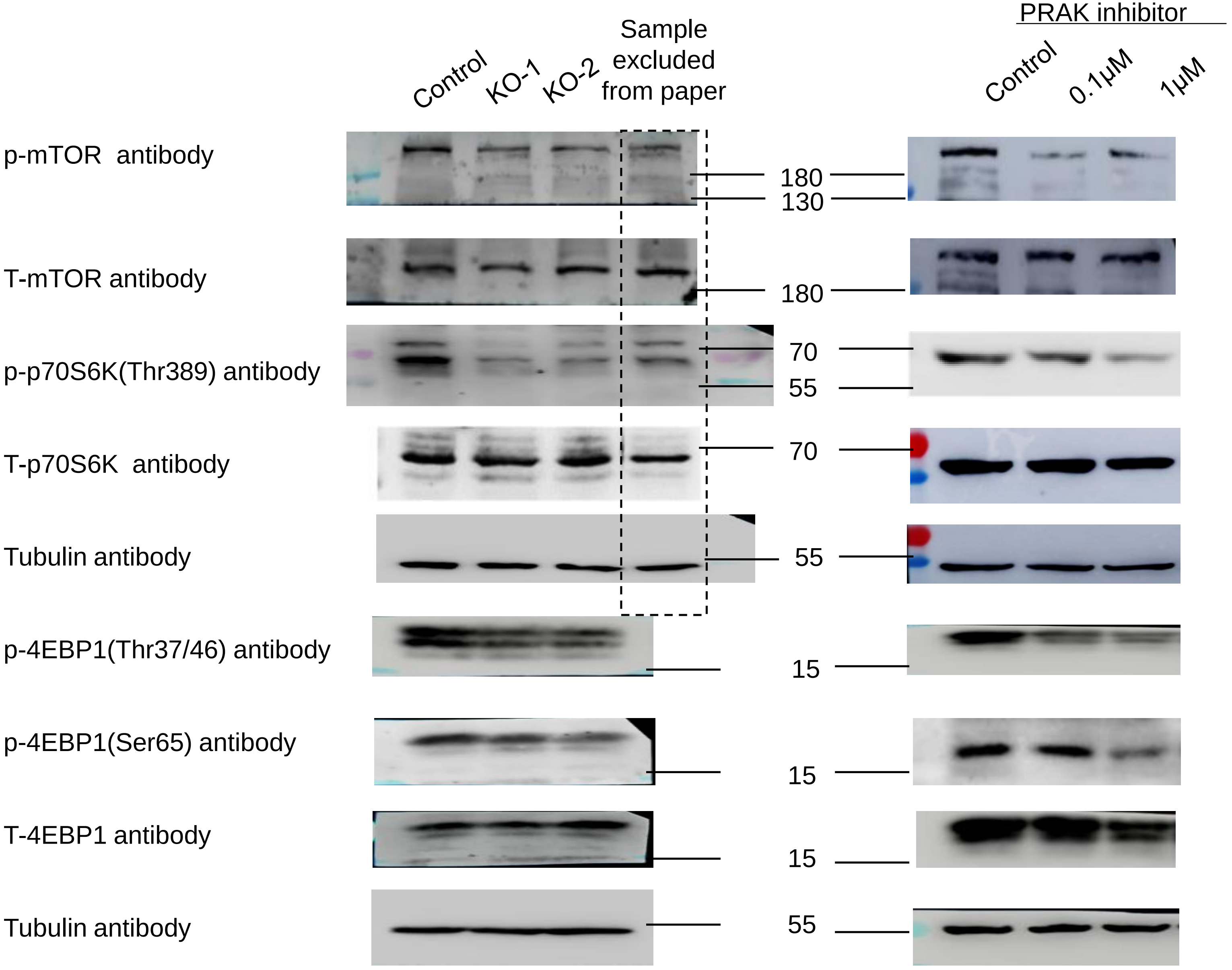
Control
KO-1
KO-2
Sample excluded from paper
PRAK inhibitor
Control
0.1μM
1μM
p-mTOR antibody
T-mTOR antibody
p-p70S6K(Thr389) antibody
T-p70S6K antibody
Tubulin antibody
p-4EBP1(Thr37/46) antibody
p-4EBP1(Ser65) antibody
T-4EBP1 antibody
Tubulin antibody
180
130
180
70
55
70
55
15
15
15
55

Figure 5F

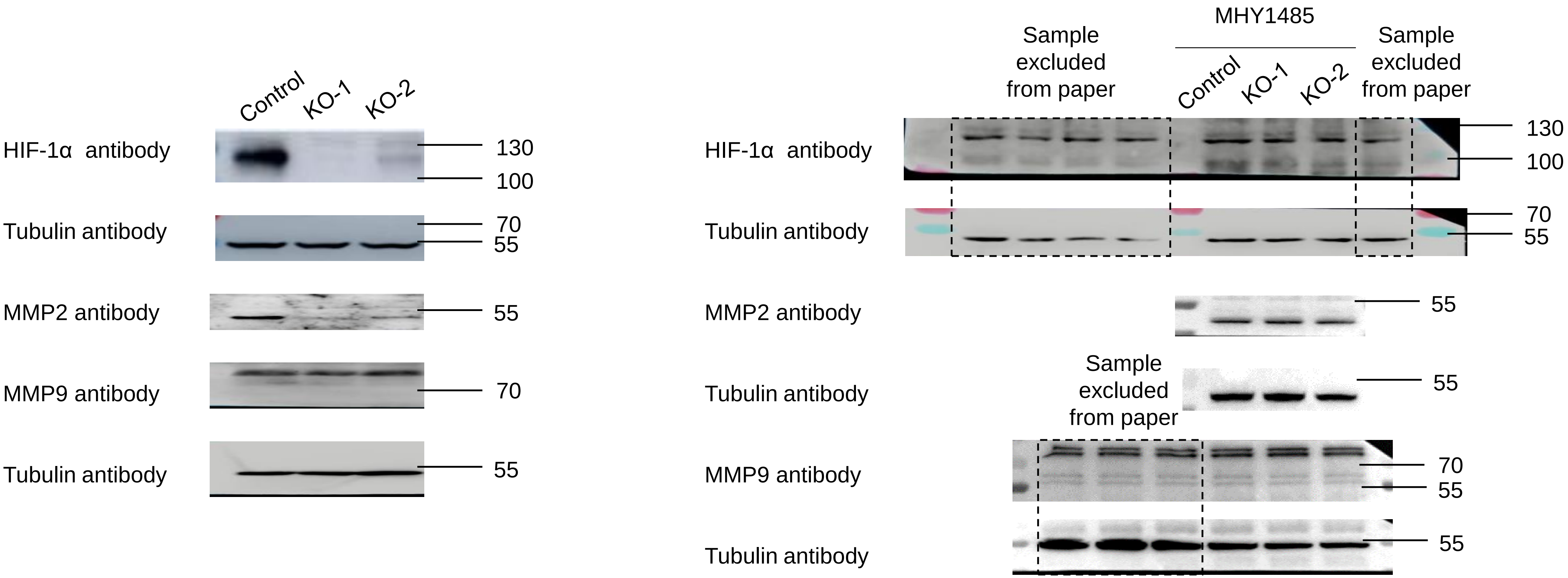
Control
KO-1
KO-2
HIF-1α antibody
130
100
Tubulin antibody
70
55
MMP2 antibody
55
MMP9 antibody
70
Tubulin antibody
55
MHY1485
Sample excluded from paper
Control
KO-1
KO-2
Sample excluded from paper
HIF-1α antibody
130
100
Tubulin antibody
70
55
MMP2 antibody
55
Tubulin antibody
55
Sample excluded from paper
MMP9 antibody
70
55
Tubulin antibody
55

Figure S1A

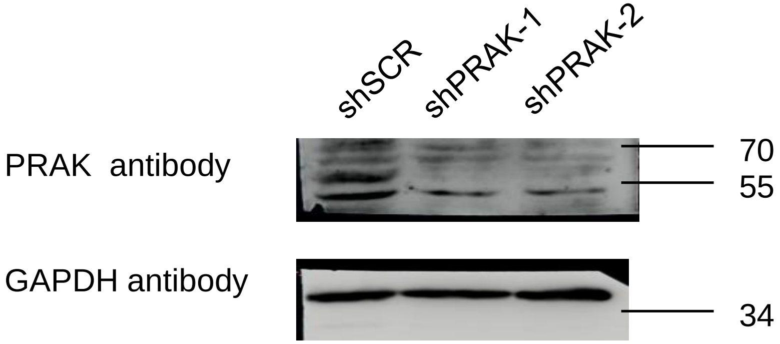

Figure S1C

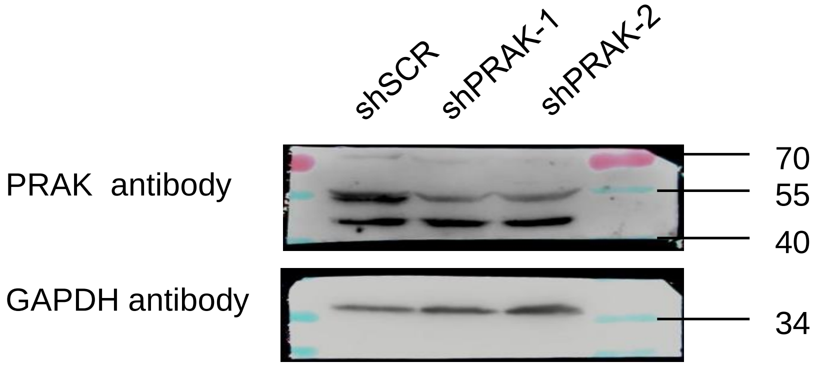

Figure S4B

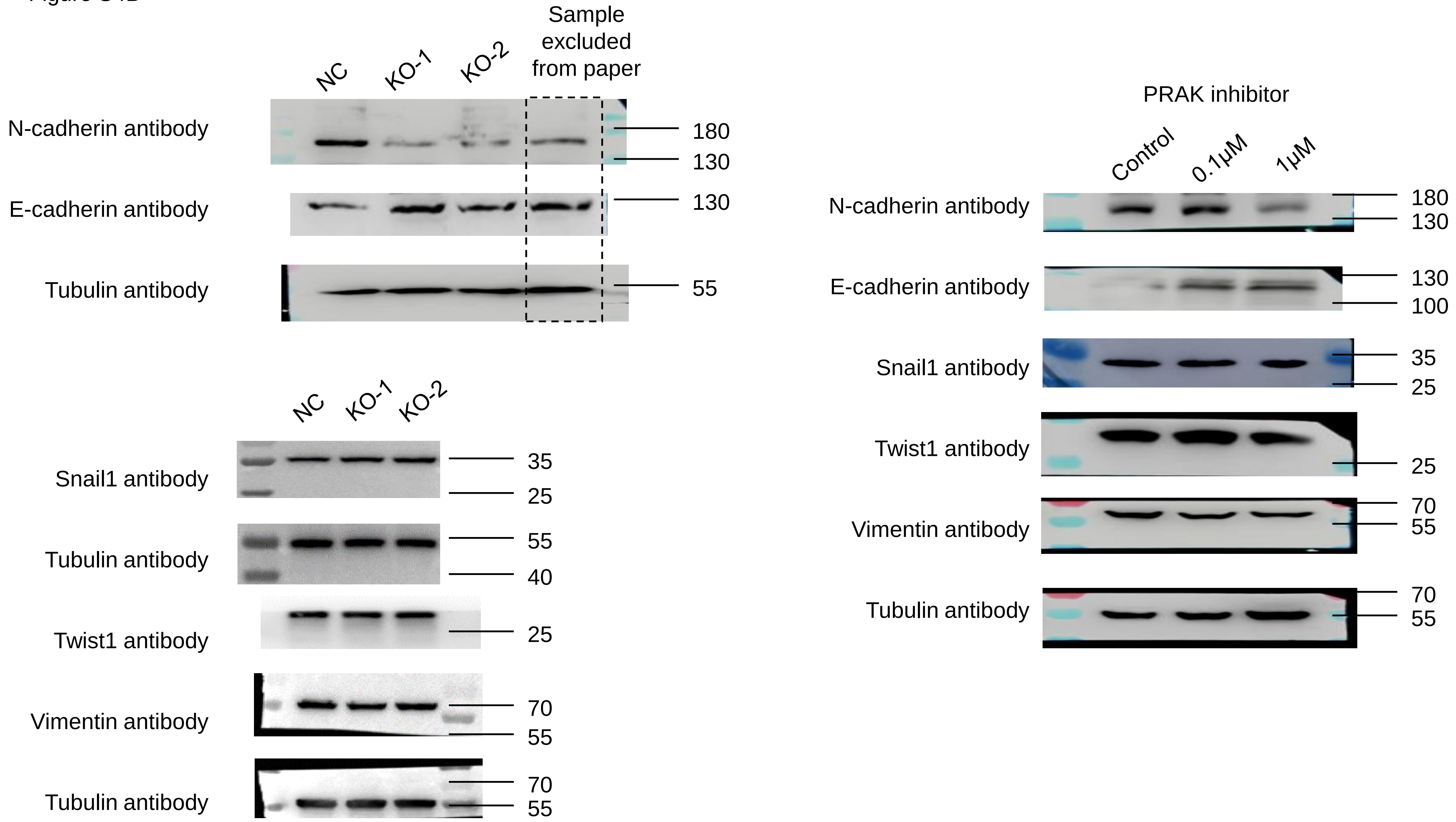

NC
KO-1
KO-2
Sample excluded from paper
N-cadherin antibody
180
130
E-cadherin antibody
130
Tubulin antibody
55
NC
KO-1
KO-2
Snail1 antibody
35
25
Tubulin antibody
55
40
Twist1 antibody
25
Vimentin antibody
70
55
Tubulin antibody
70
55
PRAK inhibitor
Control
0.1μM
1μM
N-cadherin antibody
180
130
E-cadherin antibody
130
100
Snail1 antibody
35
25
Twist1 antibody
25
Vimentin antibody
70
55
Tubulin antibody
70
55

Figure S4C

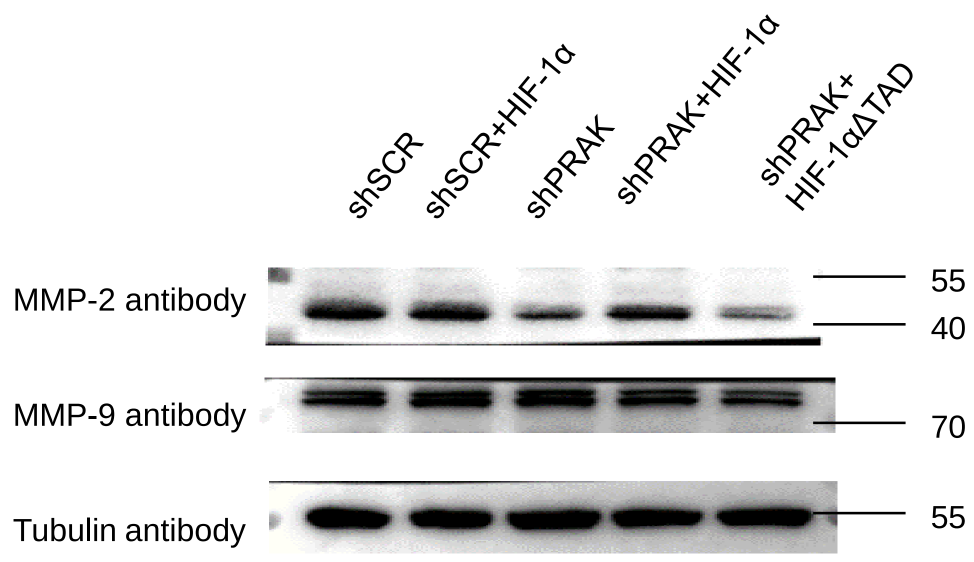
shSCR
shSCR+HIF-1α
shPRAK
shPRAK+HIF-1α
shPRAK+ HIF-1αΔTAD
MMP-2 antibody
55
40
MMP-9 antibody
70
Tubulin antibody
55

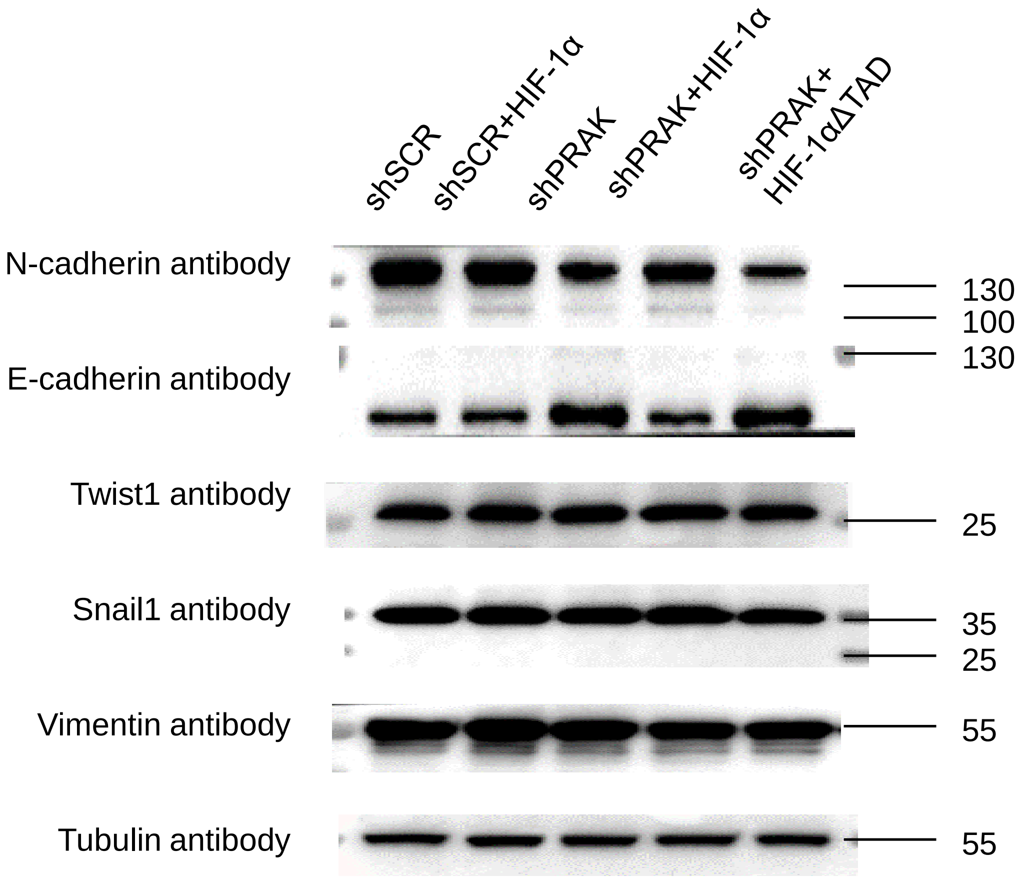
shSCR
shSCR+HIF-1α
shPRAK
shPRAK+HIF-1α
shPRAK+ HIF-1αΔTAD
N-cadherin antibody
130
100
E-cadherin antibody
130
Twist1 antibody
25
Snail1 antibody
35
25
Vimentin antibody
55
Tubulin antibody
55

Figure S5C

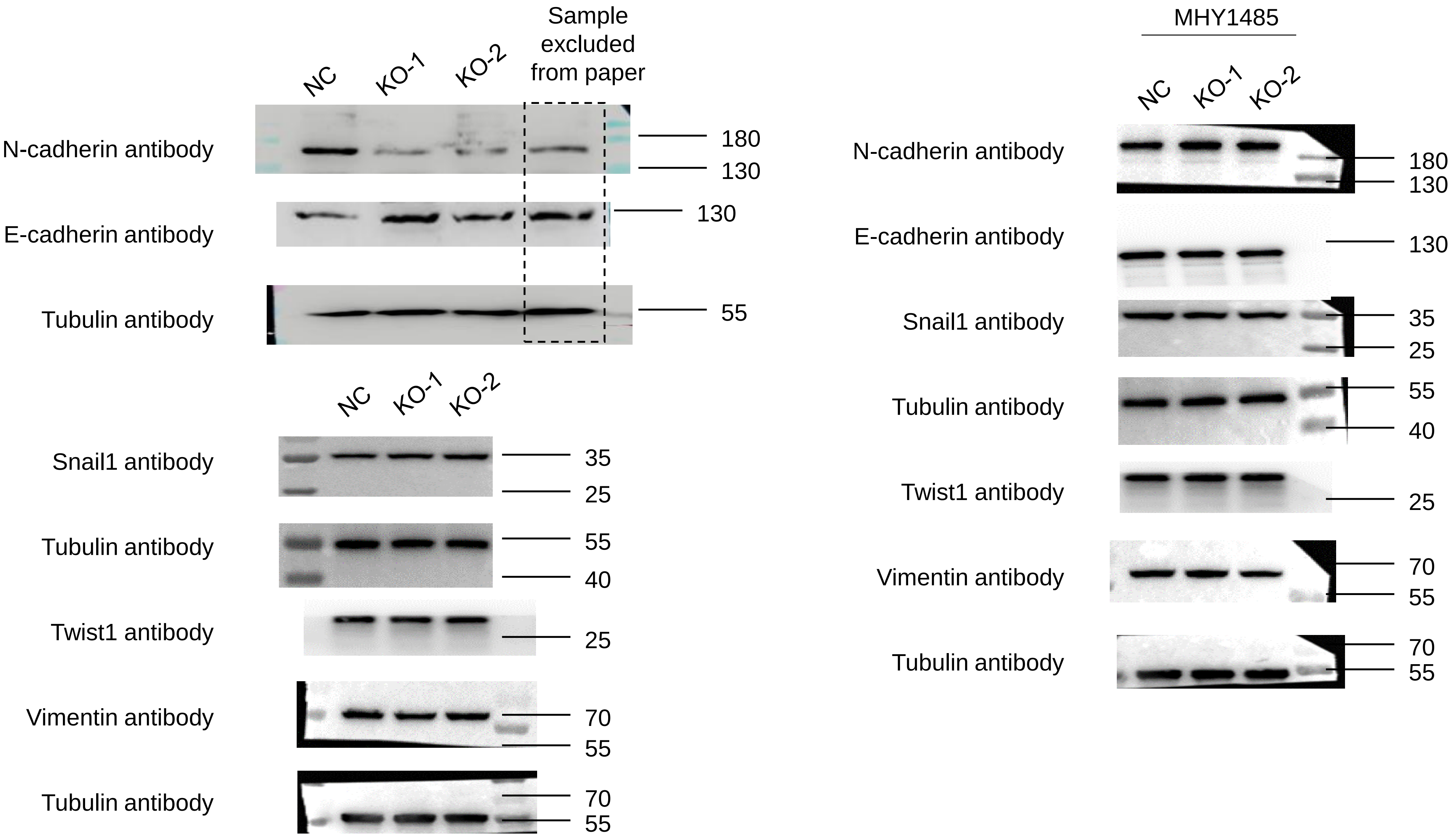

NC
KO-1
KO-2
Sample excluded from paper
N-cadherin antibody
180
130
E-cadherin antibody
130
Tubulin antibody
55
NC
KO-1
KO-2
Snail1 antibody
35
25
Tubulin antibody
55
40
Twist1 antibody
25
Vimentin antibody
70
55
Tubulin antibody
70
55
MHY1485
NC
KO-1
KO-2
N-cadherin antibody
180
130
E-cadherin antibody
130
Snail1 antibody
35
25
Tubulin antibody
55
40
Twist1 antibody
25
Vimentin antibody
70
55
Tubulin antibody
70
55
